# Searching for Mechanisms Underlying the Assembly of Calcium Entry Units: The Role of Temperature and pH

**DOI:** 10.3390/ijms24065328

**Published:** 2023-03-10

**Authors:** Barbara Girolami, Matteo Serano, Alessia Di Fonso, Cecilia Paolini, Laura Pietrangelo, Feliciano Protasi

**Affiliations:** 1 DMSI, Department of Medicine and Aging Sciences & CAST, Center for Advanced Studies and Technology, University G. d’Annunzio of Chieti-Pescara, 66100 Chieti, Italy; barbara.girolami@unich.it (B.G.); matteo.serano@unich.it (M.S.); alessia.difonso@unich.it (A.D.F.); laura.pietrangelo@unich.it (L.P.); 2 DNISC, Department of Neuroscience and Clinical Sciences & CAST, Center for Advanced Studies and Technology, University G. d’Annunzio of Chieti-Pescara, 66100 Chieti, Italy; cecilia.paolini@unich.it

**Keywords:** exercise, excitation-contraction (EC) coupling, skeletal muscle, store-operated Ca^2+^ entry (SOCE)

## Abstract

Store-operated Ca^2+^ entry (SOCE) is a mechanism that allows muscle fibers to recover external Ca^2+^, which first enters the cytoplasm and then, via SERCA pump, also refills the depleted intracellular stores (i.e., the sarcoplasmic reticulum, SR). We recently discovered that SOCE is mediated by Calcium Entry Units (CEUs), intracellular junctions formed by: (i) SR stacks containing STIM1; and (ii) I-band extensions of the transverse tubule (TT) containing Orai1. The number and size of CEUs increase during prolonged muscle activity, though the mechanisms underlying exercise-dependent formation of new CEUs remain to be elucidated. Here, we first subjected isolated extensor digitorum longus (EDL) muscles from wild type mice to an ex vivo exercise protocol and verified that functional CEUs can assemble also in the absence of blood supply and innervation. Then, we evaluated whether parameters that are influenced by exercise, such as temperature and pH, may influence the assembly of CEUs. Results collected indicate that higher temperature (36 °C vs. 25 °C) and lower pH (7.2 vs. 7.4) increase the percentage of fibers containing SR stacks, the n. of SR stacks/area, and the elongation of TTs at the I band. Functionally, assembly of CEUs at higher temperature (36 °C) or at lower pH (7.2) correlates with increased fatigue resistance of EDL muscles in the presence of extracellular Ca^2+^. Taken together, these results indicate that CEUs can assemble in isolated EDL muscles and that temperature and pH are two of the possible regulators of CEU formation.

## 1. Introduction

The role of external Ca^2+^ in skeletal muscle contractility has been overlooked for many years, as excitation-contraction (EC) coupling, the mechanism that allows activation of muscle contraction by transducing the action potential into release of Ca^2+^ from the sarcoplasmic reticulum (SR), is mechanical in skeletal muscle and does not depend on Ca^2+^ entry as in cardiac and smooth muscle [1,2,3,4,5,6]. In mechanical coupling, the alpha-1s subunit of DHPRs, voltage-gated L-type Ca^2+^ channels (also known as Cav1.1) of transverse tubules (TTs) act as a voltage sensor [5,7], which directly activates the SR Ca^2+^ release from RyR type-1 (RyR1) channels [8,9,10]. The intracellular sites that allow mechanical interaction between DHPR and RyR1 are called Ca^2+^ Release Units (CRUs) or triads, intracellular junctions formed by the association of two SR terminal cisternae with a central TT [11,12].

Recently the role that Ca^2+^ entry may play in skeletal muscle function has attracted new attention. A pathway known as excitation-coupled Ca^2+^ entry (ECCE), independent of SR store depletion and not required for EC coupling itself, was identified as a slow Ca^2+^ entry pathway through DHPRs based on a retrograde signal from RyR1 to Cav1.1 [13,14,15,16]. ECCE causes Ca^2+^ influx in response to physiological triggers [17,18]. In addition, a mechanism triggered by depletion of intracellular Ca^2+^ stores (endoplasmic/sarcoplasmic reticulum, ER and SR), known as store-operated Ca^2+^ entry (SOCE) [19,20,21,22], was also reported in skeletal muscle [23]. SOCE is a pathway mainly mediated by the interaction between (a) stromal interaction molecule-1 (STIM1), a protein placed in the ER/SR membrane, which has an intra-luminal domain that acts as Ca^2+^ sensor; and (b) Orai1, a protein that mediates Ca^2+^ release-activated (CRAC) current and is placed in external membranes or TTs [24,25,26,27,28,29,30,31,32,33,34].

The mechanism of activation of SOCE was first studied in non-excitable cells: Ca^2+^ depletion of internal ER stores induces dimerization of STIM1 and its translocation towards the plasma membrane (PM), enabling in this way STIM1 to interact and activate Orai1 Ca^2+^ channels [34,35]. In skeletal muscle, SOCE also relies on the concerted activity of STIM1 and Orai1 [25,30,36,37,38], as supported by the finding that SOCE is abolished in mice lacking STIM1 [39] and in muscle fibers from dominant-negative and muscle-specific Orai1-knockout mice [40,41]. The exact stoichiometry of the active SOCE complex is currently unclear [42,43]. Several studies hypothesized a 1:1 STIM1:Orai1 stoichiometry, with a STIM1s dimer that interacts with a pair of Orai1 C-terminal subunits [44,45,46,47]. It has also been reported that STIM1-Orai1 complexes form as a result of the interaction of oneSTIM1 dimer with one Orai1 channel [22].

The idea that impairment in SOCE can be behind muscle pathologies characterized by increased fatigability and reduced SR Ca^2+^ release [48] has recently increased the interest in this mechanism in the muscle system. Indeed, aberrant SOCE function was associated with several forms of muscle dysfunction: weakness in aging [49,50,51], oversensitivity to heat in malignant hyperthermia [52,53], and muscular dystrophy in mdx mice [54,55,56,57]. In addition, mutations in STIM1 and Orai1 have been linked to tubular aggregate myopathy (TAM), a rare condition characterized by muscle pain, cramping, weakness, and the presence of peculiar remodeling of the SR [58,59,60,61,62,63,64,65].

Some authors reported that SOCE in skeletal muscle fibers has evolved in a different mechanism that is activated in two different modes: (i) rapidly activated SOCE (within milliseconds) of limited amplitude (phasic SOCE); and (ii) a slowly activated, large-scale SOCE or chronic SOCE [64,66,67,68,69,70]. Phasic SOCE in muscle fibers is activated significantly faster than in non-excitable cells, where the process from ER store depletion to Orai1 channel activation takes tens of seconds [35,67,69,70,71,72,73]. Rapid activation of SOCE in skeletal fibers could be explained by STIM1 and Orai1 being pre-assembled in specific sites. For this reason, initially it was proposed that SOCE would occur in triads, the intracellular junctions deputed to EC coupling (see above). This hypothesis was based on the assumption that in triads SR and TT membranes, which contain respectively STIM1 and Orai1, are already associated and would easily allow rapid activation of SOCE. A STIM1 splice variant highly expressed in skeletal muscle (STIM1-long) was proposed to account for rapid SOCE activation [74,75]. Recently, some authors also suggested that slow and fast SOCE may use different pools of Orai1 channels [76].

While there was always general agreement about the important role that SOCE plays in limiting muscle fatigue [40,41,50,51,77], exactly how and where STIM1/Orai1 interact to allow Ca^2+^ entry in skeletal muscle fibers has been debated for several years. Recent experimental evidence collected in our laboratory indicated that intracellular junctions, named Ca^2+^ Entry Units (CEUs), formed by the association of SR and TT at the I band, are the most likely sites of interaction between STIM1 and Orai1 during SOCE [77,78,79,80,81,82,83]. CEUs are few and small in control conditions (the main reason that did not allow their prompt identification), but increase in number and size during exercise [77] to then disassemble following recovery [81]. CEU assembly during exercise requires: (i) remodeling of SR membranes at the I band to form stacks of flat cisternae (i.e., SR stacks); and (ii) elongation of TTs from the triad toward the Z line. This remodeling promotes enhanced STIM1 and Orai1 colocalization and increased fatigue resistance in the presence of extracellular Ca^2+^ [77]. After proposing that these junctions provide a structure to recover Ca^2+^ ions via SOCE from the extracellular space [77,78], the conclusive evidence of CEUs being a site for SOCE came with the demonstration that: (i) these junctions promote increased rate on Mn^2+^ quench, i.e., the gold-standard technique used to assess entry of divalent cations from the extracellular space [77,78,81,82]; and (ii) exercise-dependent enhancement of SOCE, due to assembly of additional CEUs, does not occur in muscle fibers lacking Orai1 [81]. Finally, as proof of principle, we also found that CEUs are constitutively assembled in muscle fibers of mice lacking CASQ1 [82], which develop quickly to SR depletion (i.e., the putative trigger of STIM1 aggregation and SOCE activation) during repetitive stimulation [84].

Together, these findings indicate that CEUs are dynamic intracellular junctions between SR and TTs that form when muscle fibers need to use external Ca^2+^ to replenish depleted SR stores, hence limiting muscle fatigue. Nevertheless, the mechanisms that promote the formation of SOCE-sites (i.e., CEUs) during exercise and disassembly during post-training recovery are still obscure. In this work, we first: (i) investigated if CEUs can assemble in the absence of blood supply and innervation in isolated extensor digitorum longus (EDL) muscles stimulated ex vivo; and then (ii) studied the influence of temperature and extracellular pH in CEU assembly, two parameters that change physiologically during exercise in vivo [85,86,87,88,89].

## 2. Results

### 2.1. Functional Calcium Entry Units Can Assemble Ex Vivo

**Figure 1:**
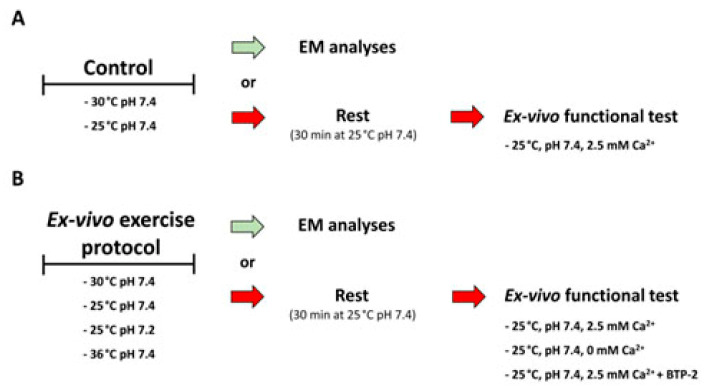
**Schematic representation of the ex vivo experimental procedures**. (**A**) Control EDL muscles were kept at rest in KH solution for 45 min (the same duration of the ex vivo exercise protocol) and then either immediately fixed for EM analysis (green arrow) or kept in a rest condition for 30 min (25 °C, pH 7.4) and then subjected to the ex vivo functional test (red arrows). (**B**) EDL muscles exercised ex vivo were either immediately fixed for EM analysis or kept in rest condition for 30 min (25 °C, pH 7.4) and then subjected to the ex vivo functional test.

We recently demonstrated that acute exercise in vivo promotes the assembly of CEUs, intracellular junctions located at the I band that mediate SOCE. CEUs are formed by the association of two distinct components: SR stacks and I band extensions of TTs [77,78,81]. To verify whether exercise-dependent assembly of CEUs can take place in the absence of blood supply and innervation, we subjected isolated EDL muscles of adult wild type (WT) mice to an ex vivo exercise protocol at 30 °C (see Section 4. Materials and Methods and scheme in Figure 1 for additional detail).

**Figure 2:**
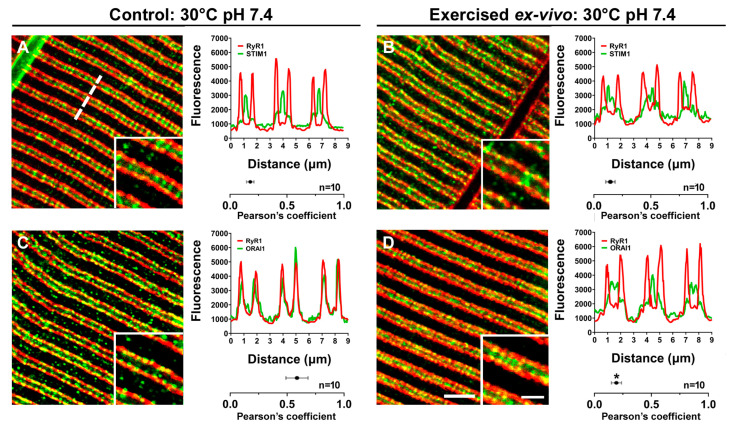
**Sarcomeric localization of STIM1 and Orai1 before and after an ex vivo protocol performed at 30 °C and pH 7.4.** Representative immunofluorescence images of EDL fibers showing RyR1 and STIM1 (**A**,**B**) and RyR1-Orai1 (**C**,**D**) double-staining. Each panel contains also a fluorescence intensity profile along three sarcomeres (see dashed line in (**A**)) and the Pearson’s correlation coefficient value, i.e., a method of measuring the covariance of pixel intensities, given as the mean ± SEM. * *p* < 0.01, compared to fibers from control mice; n = number of images analyzed. Scale bar: 2.5 µm (insets: 1 µm).

Samples from two different groups were fixed for immunofluorescence and electron microscopy (EM): (a) EDL muscles that were not electrically stimulated (controls), but simply kept in the solution at 30 °C for 45 min (the same duration of the ex vivo exercise protocol); (b) EDL muscles subjected to the ex vivo exercise protocol. To determine the subcellular co-localization between STIM1 and Orai1 under control conditions and to verify if STIM1-Orai1 colocalization increases following the ex vivo exercise protocol (as in vivo during acute exercise [77]), small bundles of EDL fibers were double-labeled in immunofluorescence experiments (Figure 2) as follows: RyR1 vs. STIM1 (Figure 2A,B) and RyR1 vs. Orai1 (Figure 2C,D).

In Boncompagni et al., 2017 [77], we showed that in resting muscle STIM1 is primarily localized throughout the entire I band region, while Orai1 has a preferential localization in TTs at the triads. Results collected in experiments performed for the present work confirmed previous findings (Figure 2): the level of Orai1 co-localization with RyR1 in the resting condition is high, as shown by Pearson’s correlation coefficient value (Figure 2C). On the other hand, in muscles exercised ex vivo (Figure 2D) a fraction of the Orai1 signal shifted toward the I band, decreasing in this way RyR1-Orai1 co-localization, as shown by peaks of fluorescence (traces in Figure 2D) and by the decreased Pearson’s correlation coefficient value (compared with Pearson’s correlation coefficient values in Figure 2C; *p* < 0.01). As the subcellular localization of STIM1 does not change following exercise (i.e., STIM1 is localized primarily throughout the I band region, also after the ex vivo exercise protocol; Figure 2B), the increased presence of Orai1 at the I band (Figure 2D) is consistent with an increased co-localization with STIM1.

**Figure 3:**
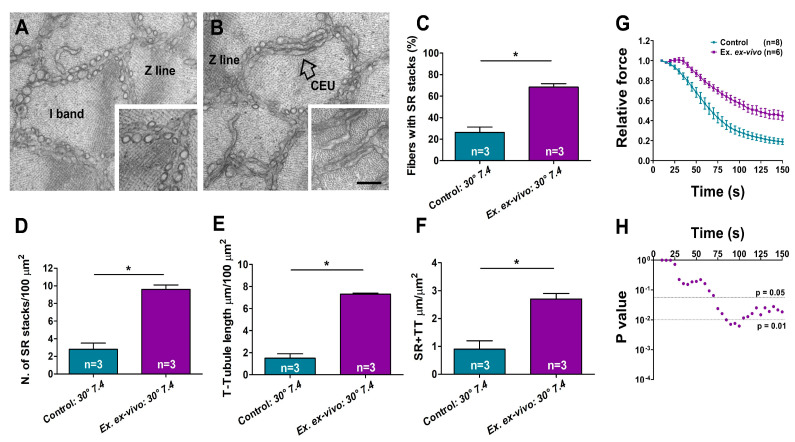
**Quantitative and functional analysis of Ca^2+^ entry units (CEUs) assembled ex vivo at 30 °C and pH 7.4**. (**A**,**B**) EM images of fibers from isolated EDLs that: (i) have rested at 30 °C and pH 7.4 for 45 min (panel (**A**)); and (ii) have been subjected to an ex vivo exercise protocol performed at 30 °C and pH 7.4 (panel (**B**)). The inset in panel A shows SR appearance at the I band; the empty arrow and the inset in panel (**B**) shows the remodeling of SR in stacks. (**C**,**D**) Percentage of fibers containing SR stacks and number of SR stacks/100 μm^2^ of section. (**E**,**F**) Extension of TTs at the I band in 100 μm^2^ of section and analysis of SR-TT contact length. (**G**) Ex vivo functional test showing time course of average relative force decay in EDL muscles previously exercised ex vivo and of controls (experiments performed at 30 °C and pH 7.4). Arrow points to the bump-phase, previously characterized in two papers by Michelucci and colleagues as the phase of activation of SOCE [81,82]. (**H**) Semi-log plot showing results of multiple unpaired *t* test followed by Tukey post hoc test. Data are shown as mean ± SEM (* *p* < 0.05). n = number of EDL analyzed. Scale bar: (**A**,**B**) = 0.1 μm; insets = 0.2 μm.

Muscles fixed and processed for EM were used to verify the presence of CEUs (Figure 3A,B). The presence of fully assembled CEUs was assessed by quantitative analysis of their two main components, i.e., SR stacks and TT extensions at the I band. We performed quantitative analysis of four different parameters that are indicative of the structural assembly of CEUs: (i) the percentage of fibers containing SR stacks (Figure 3C); (ii) the number of SR stacks/100 μm^2^ of cross-section (Figure 3D); (iii) the extension of TTs at the I band in 100 μm^2^ of cross-section (Figure 3E); and finally (iv) the extension of SR-TT contacts at the I band (Figure 3F). The data collected in Figure 3 clearly indicate that CEUs can assemble ex vivo following an exercise protocol at 30 °C and pH 7.4, as all the parameters quantified in Figure 3C–F are increased following the ex vivo exercise protocol (see also Table S1). The increased inferred colocalization of STIM1 and Orai1 at the I band (data in Figure 2) is supported by the elongation of T-tubules at the I band (Figure 3E), which underlines the translocation of Orai1 at the I band.

We previously demonstrated that the exercise-dependent assembly of CEUs increases the resistance to fatigue of EDL muscles in the presence of extracellular Ca^2+^ [77]. Here, we verified if muscles containing a greater number of CEUs (pre-exercised ex vivo) are also more resistant to fatigue than controls (Figure 3G). EDL muscles were subjected to an ex vivo functional test (see Section 4. Materials and Methods and scheme in Figure 1 for additional detail): as shown in Figure 3G, pre-exercised muscles display greater resistance to fatigue than control muscles during a fatigue protocol in which muscles were stimulated at 60 Hz, for 1 sec every 5 sec (in the presence of extracellular Ca^2+^).

### 2.2. The Assembly of CEUs Is Affected by Temperature and Extracellular pH

**Figure 4:**
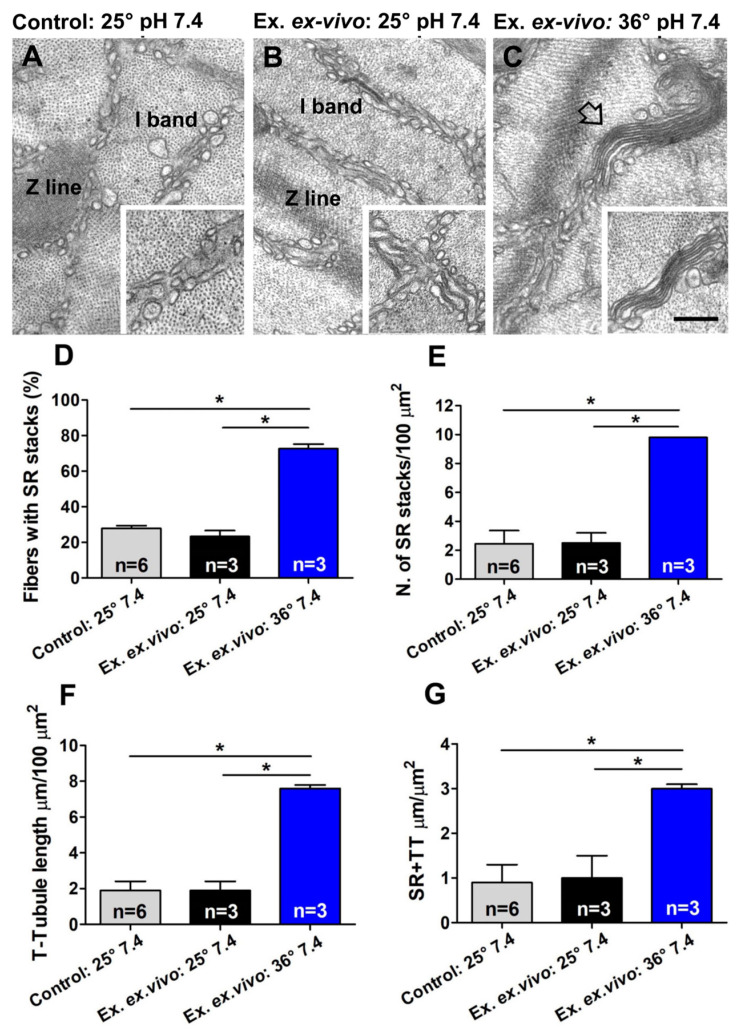
**Electron-micrographs and quantitative analysis of assembly of CEUs at 25 vs. 36 °C (pH 7.4)**. (**A**–**C**) EM images of fibers in isolated EDL muscles that: (i) have rested at 25 °C and pH 7.4 for 45 min (panel (**A**)); (ii) have been subjected to the ex vivo exercise protocol performed at 25 °C and pH 7.4 (panel (**B**)); and (iii) have been subjected to the ex vivo exercise protocol performed at 36 °C and pH 7.4 (panel (**C**)). The insets in panels (**A**,**B**) show SR appearance at the I band; the empty arrow and the inset in panel (**C**) show the remodeling of SR in stacks. (**D**,**E**) Percentage of fibers containing SR stacks and number of SR stacks/100 μm^2^ of section. (**F**,**G**) Extension of TTs at the I band in 100 μm^2^ of section and analysis of SR-TT contact length. Data are shown as mean ± SEM (* *p* < 0.05). n = number of EDL analyzed. Scale bar: (**A**–**C**) = 0.1 μm; insets = 0.2 μm.

**Figure 5:**
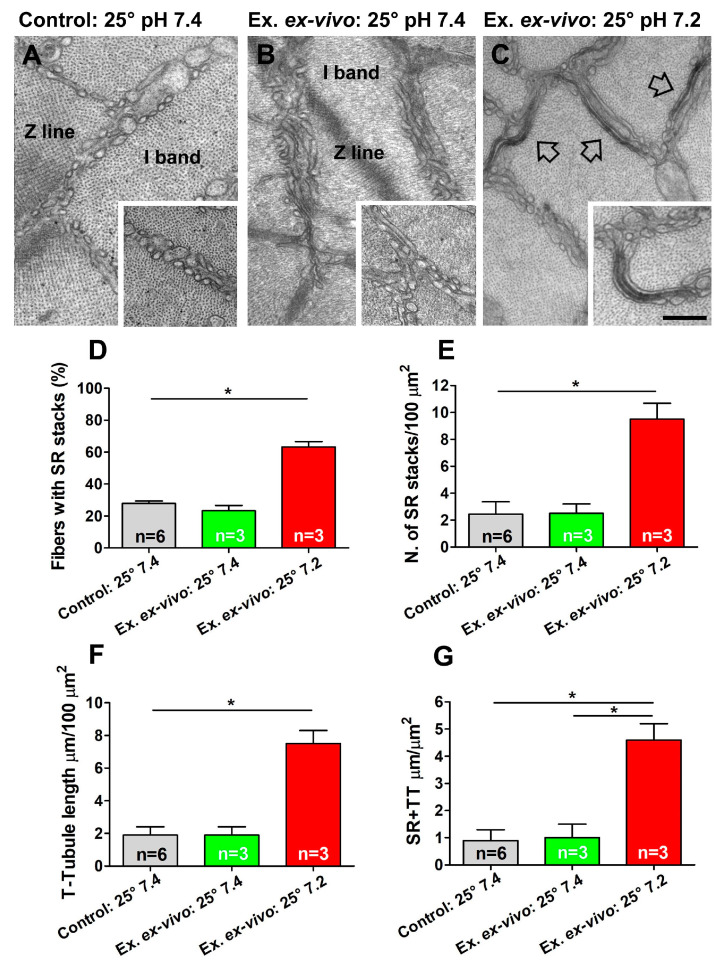
**Electron-micrographs and quantitative analysis of assembly of CEUs at pH 7.4 vs. 7.2 (25 °C)**. (**A**–**C**) EM images in isolated EDL muscles that: (i) have rested at 25 °C and pH 7.4 (panel (**A**)); (ii) have been subjected to the ex vivo exercise protocol performed at 25 °C and pH 7.4 (panel (**B**)); and (iii) have been subjected to the ex vivo exercise protocol performed at 25 °C and pH 7.2 (panel (**C**)). The insets in panel (**A**,**B**) show SR appearance at the I band; the empty arrows and the inset in panel (**C**) show the remodeling of SR in stacks. (**D**,**E**) Percentage of fibers containing SR stacks and number of SR stacks/100 μm^2^ of section. (**F**,**G**) Extension of TTs at the I band in 100 μm^2^ of section and analysis of SR-TT contact length. Data are shown as mean ± SEM (* *p* < 0.05). n = number of EDL analyzed. Scale bar: (**A**–**C**) = 0.1 μm; insets = 0.2 μm.

CEUs assembled in vivo during a fatigue protocol in which mice ran on treadmill for about 1 h [77]. Nevertheless, the mechanism underlying the SR and TT remodeling necessary to increase number and size of CEUs during exercise is unknown. Here, we tested the effect of two physiological parameters that change during exercise (Figure 4 and Figure 5), i.e., body temperature rises, and extracellular pH becomes more acidic due to the combined effect of increased CO_2_ and lactic acid production [86,87,88,89]. 

### 2.3. CEUs Assembled Ex Vivo (i.e., at Higher Temperature and Lower Extracellular pH) Promote Enhanced Resistance to Fatigue in Presence of External Ca^2+^

**Figure 6:**
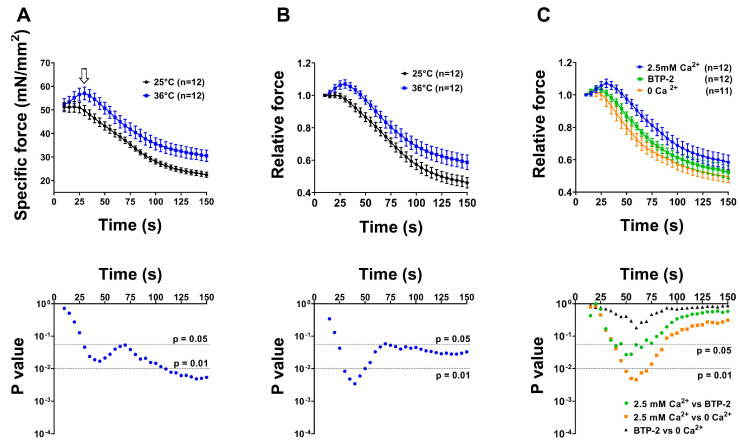
**Contractile force of EDL muscles after the ex vivo exercise protocol at 25 vs. 36 °C (pH 7.4)**. (**A**) Time course of average specific force decay during 30 consecutive frequency stimulus trains (60 Hz, 1 s, every 5 s), normalized to CSA, in EDL muscles previously ex vivo exercised at 25 °C or at 36 °C. Arrow points to the bump-phase, previously characterized in two papers by Michelucci and colleagues, as the phase of activation of SOCE [81,82]. (**B**) Data represented as relative force (normalized to the first stimulus train). (**C**) Time course of average relative force decay (in presence or absence of 2.5 mM extracellular Ca^2+^ and in a solution supplemented with 10 µM BTP-2) in EDL muscles previously subjected to the ex vivo protocol at 36 °C. Data are shown as mean ± SEM. Bottom panels represent semi-log plot showing results of multiple unpaired *t* test (first two panels) or two-way repeated measures ANOVA followed by Tukey post hoc test (right panel). n = number of EDL muscles analyzed for each condition.

**Figure 7:**
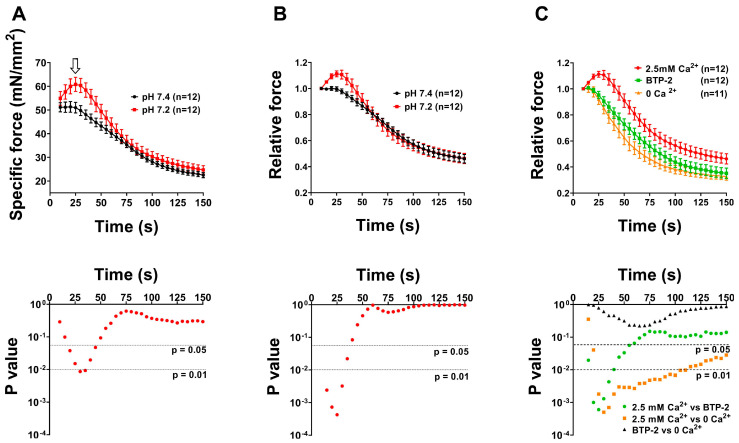
**Contractile force of EDL muscle after the ex vivo exercise protocol at pH 7.4 vs. 7.2 (25 °C)**. (**A**) Time course of average specific force decay during 30 consecutive frequency stimulus trains (60 Hz, 1 s, every 5 s), normalized to CSA, in EDL muscles previously ex vivo exercised at pH 7.4 or at pH 7.2. Arrow points to the bump-phase, previously characterized in two papers by Michelucci and colleagues as the phase of activation of SOCE [81,82]. (**B**) Data represented as relative force (normalized to the first stimulus train). (**C**) Time course of average relative force decay (in presence or absence of 2.5 mM extracellular Ca^2+^ and in a solution supplemented with 10 µM BTP-2) in EDL muscles previously subjected to the ex vivo protocol at pH 7.2. Data are shown as mean ± SEM. Bottom panels represent semi-log plot showing results of multiple unpaired *t* test (first two panels) or two-way repeated measures ANOVA followed by Tukey post hoc test (right panel). n = number of EDL muscles analyzed for each condition.

Following EM analysis shown in Figure 4 and Figure 5, we performed ex vivo functional experiments to assess the contractility of EDL muscles in the presence of either external Ca^2+^ or blocking Ca^2+^ entry by removing external Ca^2+^ (replaced by an equimolar concentration of Mg^2+^) or supplementing the solution with a compound that is frequently used to block SOCE, i.e., BTP-2 [90] (Figure 6 and Figure 7). We analyzed resistance to fatigue using a short protocol of the duration of 2.5 min (see ex vivo functional test in Section 4. Materials and Methods), in EDL muscles previously subjected to the ex vivo exercise protocol at the different conditions reported in Figure 4 and Figure 5. Specifically, we compared muscles fatigued ex vivo at 25 °C at pH 7.4 vs. 36 °C at pH 7.4 (experiments designed to test the effect of temperature on CEU assembly; structural analysis in Figure 4) and 25 °C at pH 7.4 vs. 25 °C at pH 7.2 (experiments designed to test the effect of pH; structural analysis in Figure 5). Control muscles not subjected to the ex vivo exercise protocol (analyzed with EM in Figure 4 and Figure 5) were excluded from these experiments. Results of these experiments are shown in Figure 6 and Figure 7 and summarized in the following section.

EDL muscles stimulated at 36 °C and pH 7.2 exhibited an increased ability to maintain contractile force during a high frequency fatigue protocol in comparison to those stimulated at 25 °C and pH 7.4, shown by the reduced decay of muscle force along the protocol (Figure 6A,B and Figure 7A,B). Note the bump-phase (indicatedby arrows in Figure 6A and Figure 7A), which was previously characterized in two papers by Michelucci and colleagues as the phase of activation of SOCE [81,82]. To demonstrate whether the increased fatigue resistance registered in EDL muscles exercised ex vivo at 36 °C (pH 7.4) and at 7.2 (25 °C) was due to increased Ca^2+^ entry via SOCE, we performed parallel experiments conducted using either: (i) a Ca^2+^-free solution, in which Ca^2+^ was replaced by an equimolar concentration of Mg^2+^; or (ii) a Ca^2+^-containing solution supplemented with 10 μM BTP-2 (Figure 6C and Figure 7C), an established inhibitor of SOCE [77,79,90,91,92]. These experiments indicated that the enhanced fatigue resistance of EDL muscles exercised ex vivo at 36 °C (pH 7.4) and at pH 7.2 (25 °C) containing ex vivo assembled CEUs (Figure 4 and Figure 5) was effectively due to the entry of extracellular Ca^2+^ via SOCE, as inhibition of Ca^2+^ entry results in faster decay of contractile force (Figure 6C and Figure 7C).

## 3. Discussion

### 3.1. Background

We recently found that acute exercise induces a striking remodeling of SR and TTs at the I band of sarcomeres, which leads to increased STIM1/Orai1 colocalization, enhanced Ca^2+^ entry via SOCE, and improved fatigue resistance [77,78,79,81,82,83]. These new junctions were named Ca^2+^ entry units (CEUs), dynamic entities that form during exercise to promote recovery of external Ca^2+^ to then disassemble during recovery; the mechanisms underlying their assembly are still obscure. Here, we first investigated whether CEUs can assemble in isolated muscles, in the absence of nerve and blood supply, applying an ex vivo incremental protocol to mimic the treadmill protocol previously used in vivo [77]. Then, we tested if temperature and pH, physiological parameters that change in muscle during exercise [86,87,88,89], may affect the exercise-mediated assembly of CEUs, comparing different physiological conditions: (a) 36 vs. 25 °C at pH 7.4; (b) pH 7.2 vs. 7.4 at 25 °C. After these protocols, we performed a combination of confocal and electron microscopy and ex vivo contractile experiments (in the presence or absence of external Ca^2+^) to quantify the assembly of functional CEUs in the different experimental conditions.

### 3.2. Main Findings of the Study

Data collected in the first part of this work (Figure 2 and Figure 3) suggest that CEUs can also assemble in isolated muscles during an ex vivo exercise protocol (in which temperature and extracellular pH were respectively kept at 30 °C and 7.4) without innervation and blood supply. The presence of fully assembled CEUs were verified by qualitative and quantitative EM analysis of EDL muscles excised immediately after the ex vivo exercise protocol (see Figure 1 for a scheme of the experimental procedures). These structures were analyzed in their two main components (i.e., SR stacks and elongated TTs). Increased presence of Orai1 at the I band following the ex vivo protocol (Figure 2) in immunofluorescence experiments, which implies increased co-localization with STIM1, is the consequence of TTs being more extended at the I band following the exercise protocol (Figure 3 and Table S1). Note that the I band is the sarcomeric region where CEUs assemble also in vivo [77]. The increased length of contacts between SR stacks and TTs would provide the structural framework for increased STIM1-Orai1 colocalization and for the functional interaction between them. Indeed, during the fatigue protocol in Figure 3, the force generation in the presence of external Ca^2+^ is increased in those muscles that have increased presence of CEUs.

In the second part of this study, we tested the effect of temperature and of extracellular pH on the assembly of CEUs (Figure 4, Figure 5, Figure 6 and Figure 7). Results collected indicate that the remodeling of SR in stacks and the elongation of TTs at the I band are both temperature and pH sensitive. Indeed, the EM analysis shows how number of SR stacks, elongation of TTs, and finally, the establishment of contact between SR stacks and TTs are structural parameters all influenced both by temperature and extracellular pH (Figure 4 and Figure 5 and Tables S2 and S3). The classic features of sarcotubular remodeling that emerged from the quantitative analysis were also clearly visible in EM images of cross-sections of EDL muscle fibers stimulated at 36 °C (pH 7.4) and pH 7.2 (25 °C), where the insets highlight the typical flattening of SR into stacks (EM images in Figure 4 and Figure 5).

It is also important to underline two other aspects of these findings:

- Both higher temperature (36 °C) and lower pH (7.2) promote CEU assembly independently, as: (i) in experiments comparing 25 °Cvs. 36 °C, the extracellular pH was kept in both at 7.4 (Figure 4 and Table S2); (ii) when we tested pH 7.2 vs. 7.4, temperature was kept in both at 25 °C (Figure 5 and Table S3). Yet in both conditions, formation of CEUs was greatly improved only changing one of the two parameters (Figure 4 and Figure 5 and Tables S2 and S3).
- Numeric values collected in quantitative analysis of EDL muscles stimulated at 25 °C (pH 7.4) (Figure 4 and Figure 5 and Tables S2 and S3) were not different from those of control muscles, i.e., EDL muscles held at 25 °C (pH 7.4) for 45 min and not subjected to the ex vivo exercise protocol (Figure 4 and Figure 5 and Tables S2 and S3).

Structural assembly of CEUs assessed in Figure 4 and Figure 5 by EM was followed by functional protocols aiming to determine fatigue resistance in different conditions: (i) in the presence of external Ca^2+^, (ii) in nominally Ca^2+^ free solution, and (iii) in the presence of external Ca^2+^, but in a solution supplemented with a SOCE inhibitor (BTP-2) (Figure 6 and Figure 7). The results collected indicate that muscles containing more CEUs display a greater fatigue resistance (Figure 6A,B and Figure 7A,B), which is reduced in conditions that limit Ca^2+^ entry from the extracellular space (Figure 6C and Figure 7C).

One aspect of our findings deserves additional attention: when comparing structural assembly of CEUs by EM at 30 °C and 36 °C (Figure 3 vs. Figure 4; see also Tables S1 and S2) surprisingly, we found no significant quantitative difference in the formation of SR stacks and elongation of TTs. Though, functional analysis suggests that activation of SOCE at 30 °C is not as prompt as at 36 °C, as shown by the slightly less pronounced bump-phase at the lower temperature (compare Figure 3G with Figure 6). The reason for this difference is still unclear and deserves a more in-depth investigation. Though we could speculate that while SR stacks and TT elongation are fully assembled already at 30 °C, at 36 °C either (i) part of STIM1 and Orai1 are already pre-assembled or (ii) they respond to electrical stimulation more promptly at higher temperature.

### 3.3. Our Findings in the Context of Existing Knowledge

It is well known that skeletal muscle tissue is capable of great plasticity and shows a wide spectrum of adaptations in response to mechanical and metabolic stress induced by physical exercise.

- *Changes in temperature*. STIM1-mediated SOCE has been proposed to play a critical role inthe development, contraction, fatigue resistance, and remodeling of skeletal muscle cells [39,40]. Xiao and colleagues demonstrated that heating cells induce STIM1 clustering and activation of STIM1/Orai1-mediated Ca^2+^ influx [93]. Moreover, the same authors demonstrated that temperature sensitivity of STIM1-dependent Ca^2+^ signaling can impact gene expression in immune cells in response to heat alone, suggesting that STIM1 acts as a temperature sensor. Muscle activity generates heat: the temperature of human skeletal muscle can increase from 33 °C up to 39 °C during exercise [94], raising the possibility that heat-induced Ca^2+^ influx via SOCE could play a role in skeletal muscle physiology. We have recently shown that exercise-dependent assembly of CEUs could contribute to exertional heat stroke when exercise is performed in adverse environmental conditions [92]. In the present study, the assembly of functional CEUs was favored by physiological temperature, when compared to 25 °C. In addition, functional activation of SOCE (shown by the bump-phase, pointed by arrows in Figure 6 and Figure 7) was more pronounced at 36 °C than at 25 or 30 °C.
- *Changes in pH*. Cytosolic pH in cells is tightly regulated [95], because dramatic differences in protein function and cell behavior are driven by relatively small changes in pH. Under normal physiological conditions extracellular pH in healthy tissues is maintained within a narrow range between 7.3 and 7.4, while intracellular pH is kept between 7.1 and 7.2. For example, increases in pH are permissive for growth factor–induced cell proliferation [96], cell cycle progression [97,98], and differentiation [99,100]. Regarding pH regulation of Ca^2+^ entry, SOCE-mediated platelet aggregation is dependent on extra-platelet pH [101]. In exercising muscle, both extracellular and intracellular pH can drop as low as 6.9 and 6.7, respectively [102]. Results regarding pH regulation on STIM1-Orai1 interaction are controversial, and reduction of pH has not always been associated with gain of function on SOCE. Tsujikawa and colleagues demonstrated that the Orai1/STIM1 channel is regulated by changes of both intracellular and extracellular pH [103]. Authors showed that acidic internal and external pH reduce STIM1/Orai1 interaction, whereas alkaline intracellular and extracellular pH enhance SOCE activity. Mancarella and colleagues showed that intracellular low pH caused by oxidative stress induces uncoupling of Orai1 and STIM1, thereby inhibiting ICRAC, and that intracellular high pH causes store depletion, thereby activating ICRAC [104]. Though, in our results, a change of extracellular pH from 7.4 to 7.2 greatly improved CEU assembly and SOCE activation (Figure 5 and Figure 7).

### 3.4. Final Remarks

The present work aimed to investigate which conditions could influence the formation of functional CEUs. Our structural and functional analyses demonstrate that CEU assembly (i) is a process controlled by the muscle itself, as it does not require blood supply and innervation, as demonstrated by the fact that they do form also when neural and vascular components are removed away from the skeletal muscle; and (ii) is favored by physiological temperatures and acidification of extracellular pH, as demonstrated by manipulating the extracellular milieu surrounding the isolated muscle during ex vivo contractility experiments.

Many other parameters that deserve investigation may influence and modulate the remodeling of SR and TT during exercise. For example, STIM1 was shown to be directly activated via S-glutathionylation under conditions of oxidative stress, suggesting that STIM1 is a redox sensor. Hence, STIM1 could function as a polymodal sensor of temperature, ER Ca^2+^, and oxidative stress [105]. Several reactions occurring in muscle during exercise change the intracellular micro-environment, as (a) decrease in ATP/ADP ratio and increase in levels of inorganic phosphate; (b) repetitive activation-relaxation cycles of contractile machinery, which activate mechano-sensors in contractile filaments and cytoskeleton [106,107].

These events may also play a role in membrane remodeling during CEU assembly, even though we have not yet tested this hypothesis. See also Protasi et al., 2021 [83], for additional discussion of the role that proteins involved in TT biogenesis and membrane-bending may play [108,109,110,111].

## 4. Materials and Methods

### 4.1. Animals

All experiments were conducted according to the Directive of the European Union 2010/63/UE and were approved by the Animal Ethical Committee of the University of Chieti-Pescara and by Italian Ministry of Health (n. 1202/2020-PR).

Wild type (WT) C57bl/6 male mice were housed in microisolator cages at 20 °C in a 12 h light/dark cycle and provided free access to standard foodand water. All animals were sacrificed by cervical dislocation at 4 months of age, as approved by the Italian D. lgs. n.26/2014.

### 4.2. In Vitro Experiments

Extensor digitorum longus (EDL) muscles were excised from euthanized 4-month-old male WT mice and subjected to invitro contraction experiments using the Aurora Muscle Physiology System (1200A: Isolated Muscle System, Aurora Scientific, ON, Canada). Intact excised EDL muscles were attached to a servo motor and force transducer (model 1200A, Aurora Scientific, ON, Canada) and stimulated using two platinum electrodes in a chamber continuously perfused with Ringer’s solution as previously described [77]. Before starting each experiment, optimal stimulation and muscle length (L0) were determined using a series of 1 Hz twitch stimulation trains while stretching the muscle to a length that generated maximal force (F0). After establishing L0, muscles were first equilibrated using three tetani (0.5 s, 150 Hz) given at 1 min intervals and then ex vivo exercised to induce CEU assembly, as follows.

- Ex vivo exercise protocol (Ex. ex vivo). In order to evaluate the role of temperature and pH in the ex vivo assembly of CEUs induced by the incremental fatigue protocol, the above-mentioned protocol was carried out using Ringer’s solution at different temperatures (25 °C, 30 °C, or 36 °C) and two different pH levels (7.4 or 7.2). The experimental protocol consisted of4 steps of tetanic stimulus train: (i) 25 consecutive 0.5 s stimulus trains at 80 Hz of frequency applied every 25 s; (ii) 35 consecutive 0.5 s stimulus trains at 80 Hz of frequency applied every 20 s; (iii) 45 consecutive 0.5 s stimulus trains at 80 Hz of frequency applied every 15 s; (iv) 55 consecutive 0.5 s stimulus trains at 80 Hz of frequency applied every 10 s. EDL muscles were then directly intended for electron microscopy (EM) or subjected to an ex vivo functional test.
- Ex vivo functional test. After the ex vivo exercise protocol, muscles were kept in Ringer’s solution at 25 °C, pH 7.4, for 30 min to allow muscle recovery. For a schematic representation of the ex vivo procedures, see Figure 1. Then, EDL muscles were subjected to an ex vivo functional test consisting of 30 consecutive stimulus trains at 60 Hz of frequency (each pulse having a duration of 1 s) applied every 5 s. To determine the relative contribution of extracellular Ca^2+^ entry, experiments were conducted also in the presence of 10 µM BTP-2, an established inhibitor of SOCE [90], or in the absence of external Ca^2+^. Specific force (mN/mm^2^) was calculated by normalizing the absolute force (mN) to the cross-sectional area (CSA, mm^2^) obtained as the following formula: muscle wet weight (mg)/L0 (mm) × 1.06 (mg/mm^3^).

### 4.3. Immunofluorescence Labeling and Confocal Microscopy (CM)

EDL muscles were dissected from sacrificed mice, mounted in the above described system for ex vivo experiments, and subjected to the ex vivo exercise protocol or not (control muscles) at 30 °C. EDLs were then fixed in 2% paraformaldehyde in phosphate buffered saline (PBS) for 20 min at room temperature (RT). Small bundles of fixed fibers were (a) permeabilized for 30 min in 10% goat serum and 0.5% Triton X-100 PBS/BSA solution; (b) blocked for 1 h in PBS containing 10% goat serum; (c) washed 3 times for 10 min in PBS/BSA 1% solution; (d) incubated overnight at 4 °C in primary antibody diluted in PBS/BSA 1%; washed 3 times in PBS; (e) incubated with the secondary antibody for 1 h at RT; and (f) washed 3 times in PBS/BSA 1% solution before being mounted on coverslips with anti-bleach media. Primary antibodies used (a) mouse monoclonal anti-RyR1/RyR3 (34C antibody, 1:30, Developmental Studies Hybridoma Bank, IA, USA); (b) rabbit polyclonal anti-stromal-interacting molecule-1 (STIM1) (1:100, Sigma Aldrich, St. Louis, OH, USA); and (c) rabbit polyclonal anti-Orai1, (1:20, Thermo Scientific, Waltham, MA, USA). Secondary antibodies used (a) Cy5-labeled goat anti-mouse IgG (1:50); or (b) Cy3-labeled goat anti-rabbit (1:200) [112]. All secondary antibodies were from Jackson ImmunoResearch Laboratories (West Grove, PA, USA). Specimens were viewed and imaged using a scanning laser confocal microscope (LSM 800 Carl Zeiss, Germany) interfaced with an inverted Zeiss Axio Observer microscope. Fluorescence image profiles and co-localization were obtained from ZEN blue image analysis software (Carl Zeiss, Germany).

### 4.4. Preparation of Samples for EM

EDL muscles for ultrastructural analysis were prepared for EM after being subjected to invitroexperiments (see above). Muscles were pinned on Sylgard dishes and fixed at RT in 3.5% glutaraldehyde in 0.1 M sodium cacodylate (NaCaCO) buffer (pH 7.2) and stored in the fixative solution at 4 °C until the embedding procedure. Fixed muscles were then post-fixed, stained en-block, and embedded in epoxy resin as previously described [112,113]. Briefly, for standard EM analysis, fixed muscle samples were post-fixed for 1–2 h in 2% OsO_4_. For transverse tubule (TT) staining in EM, specimens were post-fixed in a mixture of 2% OsO_4_ and 0.8% potassium ferrocyanide (K_3_Fe(CN)_6_) for 1–2 h followed by a rinse with 0.1 M NaCaCO buffer with 75 mM CaCl_2_ and then further processed. Potassium ferrocyanide precipitate within the TT network is visualized as an electron-dense dark precipitate in EM images [77].

EM ultra-thin sections (∼50 nm of thickness) were cut from embedded samples (either with standard protocol orwith TT staining) using a Leica Ultracut R microtome (Leica Microsystem, Wien, Austria) with a 45° Diatome Ultra diamond knife (Diatome, Biel, Switzerland) and stained with uranyl acetate replacement and lead citrate. Sections were viewed at 60 kV using a FP 505 Morgagni series 268D transmission electron microscope (FEI Company, Brno, Czech Republic), equipped with a Megaview III digital camera (Olympus Soft Imaging Solutions, Munster, Germany) and Soft Imaging System.

### 4.5. Quantitative Analysis by EM

For all quantitative EM analyses, micrographs of non-overlapping regions were randomly collected from transverse sections of internal areas of fast-twitch EDL muscle fibers, as described previously [77]. The following ultrastructural parameters were evaluated:

- *SR stacks.* Incidence of fibers presenting SR stacks (expressed as percentage) and number of SR stacks (per 100 μm^2^ of section) were determined in micrographs collected from EDL muscle fibers in transverse sections. In each specimen, 15–20 fibers were analyzed, and in each fiber, 5 micrographs were taken at 28,000× magnification.
- *Non-triadic TT network at the I band.* We determined both (i) the extension of the SR in close association with the TT, and (ii) the total network of the TT at the I band of sarcomere. The non-triadic TT network was evaluated in micrographs collected from EDL muscle fibers either stained or not with ferrocyanide in transverse sections and reported as average number per area of section (100 μm^2^). In each specimen, 15–20 fibers were analyzed, and in each fiber, 5 micrographs were taken at 28,000× magnification.

### 4.6. Statistical Analyses

Statistical analyses were determined using PRISM 9 (GraphPad Software, San Diego, CA, USA) and Microsoft Excel (Microsoft Office, Redmond, WA, USA). Significance was evaluated using Chi-square, ANOVA, and t-tests for EM analysis. The significance of ex vivo experiments was evaluated using two-way ANOVA followed by Tukey’s post hoc test for pairwise comparisons of more than two groups or multiple *t* tests followed by Tukey’s post hoc test. In all cases, data are shown as mean ± SEM and differences are considered statistically significant at *p* < 0.05.

## Data Availability

Not applicable.

## Supplementary Materials

The following supporting information can be downloaded at: https://www.mdpi.com/article/10.3390/ijms24065328/s1.

## Abbreviations

CEU: Ca^2+^ Entry Unit; EC coupling: excitation-contraction coupling; EDL: extensor digitorum longus; EM: electron microscopy; SR: sarcoplasmic reticulum; STIM1: stromal interaction molecule-1; SOCE: store-operated Ca^2+^ entry; TT: transverse tubule; WT: wild type.
