# Supplementary material for: Searching for Mechanisms Underlying the Assembly of Calcium Entry Units: The Role of Temperature and pH
Source: Int J Mol Sci. 2023 Mar 10;24(6):5328. doi: 10.3390/ijms24065328 (PMC10049691; doi:10.3390/ijms24065328)
Supplement: Supplementary file 1 [file ijms-24-05328-s001.zip › ijms-2151275-supplementary.pdf]

SUPPLEMENTAL MATERIAL

Table S1. *Quantitative analysis by Electron Microscopy (EM) of SR stacks and TT-extension at the I band in EDL fibers from Control (at 30°C and pH 7.4) and Exercised ex-vivo at 30°C and pH 7.4. Data contained in this Table were used to construct the bar graphs in Fig. 3 C-F.*

	**Fibers with SR stacks (%)**	**N. of SR stacks/ 100 μm^2**	**T-Tubule length μm/100 μm^2**	**SR+TT μm/μm^2**
**Control: 30° 7.4**	26.3 ± 5.0	2.8 ± 0.7	1.5 ± 0.4	0.9 ± 0.3
**Ex. *ex-vivo*: 30° 7.4**	68.4 ± 3.2*	9.6 ± 0.5*	7.3 ± 0.1*	2.7 ± 0.2*

Data are shown as mean ± SEM ; $^*p<0.05$. Samples size: 3 mice for each group.

Table S2. *Quantitative analysis by EM of SR stacks and TT-extension at the I band in EDL fibers from Control (at 25°C and pH 7.4), Exercised ex-vivo at 25°C and pH 7.4 and Exercised ex-vivo at 36°C and pH 7.4. Data contained in this Table were used to construct the bar graphs in Fig. 3 D-G.*

	**Fibers with SR stacks (%)**	**N. of SR stacks/ 100 μm²**	**T-Tubule length μm/100 μm²**	**SR+TT μm/μm²**
**Control: 25° 7.4**	27.8 ± 1.4	2.4 ± 0.9	1.9 ± 0.5	0.9 ± 0.4
**Ex. *ex-vivo*: 25° 7.4**	23.3 ± 3.3*	2.5 ± 0.7*	1.9 ± 0.5*	1.0 ± 0.5*
**Ex. *ex-vivo*: 36° 7.4**	72.6 ± 2.6*	9.8 ± 0.1*	7.6 ± 0.2*	3.0 ± 0.1*

*Data are shown as mean ± SEM; *p < 0.05 vs. Control: 25° 7.4. Samples size: 6 Control mice, 3 mice for both Ex. ex-vivo:25° 7.4 and Ex. ex-vivo: 36° 7.4 groups.*

Table S3. *Quantitative analysis by EM of SR stacks and TT-extension at the I band in EDL fibers from Control (at 25°C and pH 7.4), Exercised ex-vivo at 25°C and pH 7.4 and Exercised ex-vivo at 25°C and pH 7.2. Data contained in this Table were used to construct the bar graphs in Fig. 3 D-G.*

	**Fibers with SR stacks (%)**	**N. of SR stacks/ 100 μm²**	**T-Tubule length μm/100 μm²**	**SR+TT μm/μm²**
**Control: 25° 7.4**	27.8 ± 1.4	2.4 ± 0.9	1.9 ± 0.5	0.9 ± 0.4
**Ex. *ex-vivo*: 25° 7.4**	23.3 ± 3.3	2.5 ± 0.7	1.9 ± 0.5	1.0 ± 0.5
**Ex. *ex-vivo*: 25° 7.2**	63.3 ± 3.3*	9.5 ± 1.2*	7.5 ± 0.8*	4.6 ± 0.6*

*Data are shown as mean ± SEM; *p < 0.05 vs. Control: 25° 7.4. Samples size: 6 Control mice, 3 mice for both Ex. ex-vivo:25° 7.4 and Ex. ex-vivo: 25° 7.2 groups.*
